# Proteolytic profile of larval developmental stages of *Penaeus vannamei*: An activity and mRNA expression approach

**DOI:** 10.1371/journal.pone.0239413

**Published:** 2020-09-18

**Authors:** Liliana Rojo-Arreola, Fernando García-Carreño, Rogelio Romero, Luis Díaz Dominguez

**Affiliations:** 1 CONACyT-Centro de Investigaciones Biológicas del Noroeste, México City, México; 2 Centro de Investigaciones Biológicas del Noroeste, México City, México; Uppsala Universitet, SWEDEN

## Abstract

In arthropods, the cleavage of specific proteins by peptidases has pivotal roles in multiple physiological processes including oogenesis, immunity, nutrition, and parasitic infection. These enzymes are also key players in the larval development, and well-described triggers of molting and metamorphosis. In this work the peptidase complement throughout the larvae development of *Penaeus vannamei* was quantified at the transcript and activity level using qPCR and fluorogenic substrates designed to be hydrolyzed by class-specific peptidases respectively, providing a detailed identification of the proteolytic repertoire in *P*. *vannamei* larvae. Significant changes in the peptidase activity profile were observed. During the lecithotrophic naupliar instars, the dominant peptidase activity and expression derive from cysteine peptidases, suggesting that enzymes of this class hydrolyze the protein components of yolk as the primary amino acid source. At the first feeding instar, zoea, dominant serine peptidase activity was found where trypsin activity is particularly high, supporting previous observations that during zoea the breakdown of food protein is primarily enzymatic. At decapodid stages the peptidase expression and activity is more diverse indicating that a multienzyme network achieves food digestion. Our results suggest that proteolytic enzymes fulfill specific functions during *P*. *vannamei* larval development.

## 1. Introduction

Peptidases are hydrolases that cleave peptide bonds within protein chains. They are classified according to the catalytic mechanism and the amino acids involved in substrate hydrolysis as serine, cysteine, aspartic and metallo-peptidases [1]. In arthropods, the cleavage of specific proteins by peptidases has pivotal roles in multiple physiological processes including oogenesis, immunity, metamorphosis, larval development, nutrition and parasitic invasion [2–6]. Therefore peptidases (also referred to as proteases or proteolytic enzymes), are strongly considered as targets for parasite and insect control by interfering with their activity [7].

The relevance of proteolytic enzymes in arthropod larval development has been mostly described in economically relevant insects, for which peptidases are well-described triggers of molting and metamorphosis [5, 8], as well as partakers in fat body cell dissociation and tissue remodeling [9]. For decapod crustaceans, descriptions of peptidases in larvae are focused on enzymes participating in food digestion like trypsin and chymotrypsin [10, 11]. Profiling of expressed peptidases during larval development demonstrated a differential expression depending on the enzyme and larvae stage [12–16] suggesting peptidases as partakers in decapod larvae development.

**Fig 1:**
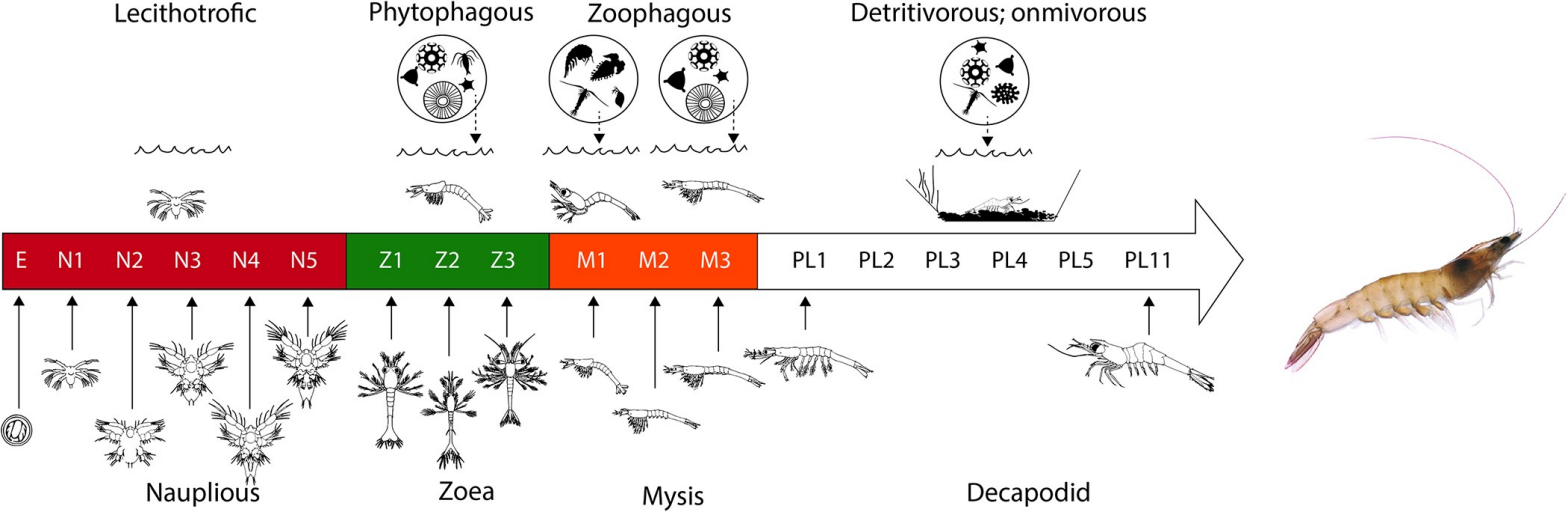
Larval developmental stages of *Penaeus vannamei*. Sub-stages, feeding habits and habitats are indicated.

*Penaus vannamei* (syn. *Litopenaeus vannamei*) is a Penaeid shrimp distributed in tropical marine environments of the Eastern Pacific coast of North, Central, and South America. Penaeid shrimps undergo a biphasic life cycle, meaning pelagic larval stages known as nauplius, zoea, mysis followed by benthonic decapodid, juvenile and adult stages. Larvae develops gradually through a series of molts occurring within a relatively short time-frame (11–17 days depending on the temperature) [17], each stage presents anatomical, physiological and ecological adaptations that fulfill its locomotive, behavioral, and feeding habits [18, 19] (Fig 1). Albeit *P*. *vannamei* is a key species for the aquaculture industry, and its rearing in captivity is highly successful, descriptions of the molecular mechanisms of many important physiological processes including larvae development are rather scarce. However, proper management of natural stocks, ecology, genetics, and technification of culture methods require a profound understanding of the developmental biology of the species.

In this work, the proteolytic profile throughout larval development of *P*. *vannamei* was assessed by means of transcripts and activity. We quantified the gene expression of 14 annotated shrimp peptidases by qPCR. Since changes in mRNA abundance do not necessarily correlate with the corresponding mature protein products, the proteolytic activity was also assessed using fluorogenic substrates designed to be hydrolyzed by serine-, cysteine-, aspartic-, and metallo-peptidases and the specific activity was confirmed by the use of class-specific inhibitors.

## 2. Materials and methods

### 2.1. Sample collection and preservation

*P*. *vannamei* samples were donated by Larvas GranMar SA de CV, San Juan de la Costa, BCS, Mexico. Samples were obtained by filtering water from the culture ponds containing larvae at each specific larval stage determined by microscope examination when at least 80% of the specimens reach the target developmental stage. A pool of ca 90 mg of larvae was sampled, washed with sterile marine water and collected into 1.6 mL cryotubes. Eight samples of each stage were obtained starting from embryos (8 hours post-spawning) (E), then in the larvae instars nauplius (N), zoea (Z), Mysis (M) and decapodid (D). Samples were immediately submerged into liquid nitrogen or RNAlater™ (depending on the intended analysis) for transportation and stored at -80°C.

### 2.2. RNA isolation, cDNA synthesis, and primer design

Total RNA was isolated from 4 replicates of 90 mg of whole larvae pools at each substage, using the Trizol™ method following the manufacture´s instructions. To remove the contaminating genomic DNA, total RNA was treated with DNase I and further purified using phenol-chloroform extraction, followed by sodium acetate and cold absolute ethanol precipitation, and 75% ethanol wash. The success of the DNase treatment was assessed by PCR, the absence of contaminating DNA resulted in no amplification bands. The concentration and purity of RNA were measured using a nano-spectrophotometer (absorbance at 260 nm and 260/280 nm ratio). The quality and integrity of the RNA were analyzed in 1% agarose gel electrophoresis.

Complementary DNA (cDNA) was synthesized from each replicate using the Reverse Transcription System kit (Promega). The reaction mixture consisted of final concentrations of 5 mM MgCl_2_ (4 μL), 1X RT Buffer, 0.5 mM dNTP mixture, Ribonuclease Inhibitor RNasin (20 U), AMV Reverse Transcriptase (0.7 μL), Oligo (dT) Primer (0.5 μg), DNA-free RNA (2.3 μg) and nuclease-free water to a final volume of 20 μL. The reverse transcription reaction was done in a thermocycler for 1 h at 42°C, followed by 5 min at 95°C, and 4 min at 4°C. The cDNA was taken to a final volume of 50 μL using nuclease-free water and stored at −20°C.

### 2.3. Peptidase transcript selection and primer design

The selection of the peptidases for which the gene expression was quantified is based on three main criteria: the existence of reports on activity and/or transcript in *P*. *vannamei*; the relevance of homologous peptidases in larvae development from other Arthropods and; the curation level in the UniProt database. Only UniProt entries having experimental evidence at protein or transcript level and/or inferred by homology were selected. Sequences annotated as preliminary data derived from whole-genome shotgun experiments were not considered for the analysis. Following these criteria, 14 peptidases were selected for transcript quantification during *P*. *vannamei* larval development (Uniprot accession numbers in Table 1).

**Table 1:** List of peptidase transcripts quantified by qPCR. Uniprot or GenBank accession numbers, primer sequences, amplicon size and standard curve properties are included.

Gen/UNIPROT accession no.	Forward and reverse primer sequence (3´–5´)	Ta	Amplicon size (bp)	Slope	R^2^	Efficiency (%)
Cathepsin L/Q27759	F–GAGCCTCTCAGAGCAGAACC	58	159	-3.578	0.999	95.2
	R–CGACACTTACCGTCCTGAGC					
Cathepsin L2/O46153	F–GTGTACTCCGACAAGACCTG	55	105	-3.576	0.999	95.2
	R–GTTCTTGACCAGCCAGAAG					
Cathepsin B/D8X153	F–TCCACAGTAAGGGCAAGAGC	57	207	-3.455	0.996	97.4
	R–CAGGGAGCAATCTCATAGGG					
Cathepsin C/B8XGG3	F–AACTGTGGCTCCTGCTATGC	57	172	-3.618	0.997	94.5
	R–CATACCTGCCAGCGATAAGG					
Calpain B/C6KE09	F–GTCACGCCTACTCCCTTACG	54	168	-3.359	0.998	99.2
	R–ATCCCATCTCCTCCTTCTCC					
Carboxypeptidase B/Q20AS8	F–CGTGACCTACATGCTGAACG	58	163	-3.621	0.994	94.4
	R–AGGAGAACCGTTGTCAGAGC					
Serine protease/Q6UKI3	F–CGACTGGTCACACTTTGC	52	192	-3.103	0.981	105.0
	R–TAAACACGACGTCTCTCTCC					
Trypsin 1/ Q9TY16	F–CATGAACAACCCCGATTACC	52	187	-3.723	0.999	92.8
	R–GCGAACGTTGTCATTGAAGC					
Trypsin 2/H6WSS5	F–CGACGACTTTGATAATCCCAGC	58	275	-3.557	0.999	95.5
	R–AGCTGCCTCCTTCAGTGAGAGC					
Trypsin 3/O62562	F–CAGAACGACATTGACGACTCC	56	174	-3.309	0.996	100.3
	R–GTACACGCCAGGGTAGTTGG					
Chymotrypsin BI/Q00871	F–CGCCCTTCCGACTCTGCCAGC	57	142	-3.672	0.998	93.6
	R–TGCTCTTGCCGCCGGTGCCGTCG					
Chymotrypsin BII/O18488	F–GCCGCCCTCTTGACAGCG	57	143	-3.619	0.996	94.5
	R–TCCCTTACCTCCTTCGGAGTCA					
Metalloendopeptidase/Q20AS7	F–ACCATCGGAGGCAAGCAGA	57	197	-3.350	0.994	99.4
	R–TGCCAGTAGGTGTCCTTGTTGA					
Cathepsin D/A0A3R7SR07	F–AATGGTCAGTGGACTGCAAC	57	155	-3.3	0.9962	100.5
	R–AACATCCAGGCCAATGAAGC					
Ubiquitin/A0A023H494	F–GGGAAGACCATCACCCTTG	60	146	-3.113	0.994	104.8
	R–TCAGACAGAGTGCGACCATC					

The design of qPCR primers was based on annotated sequences of *P*. *vannamei* peptidases (Table 1), each primer set was designed following the framework suggested by Bustin and Huggett [20], primer specificity was verified by Sanger sequencing and by the presence of a single peak revealed in melt curves. To determine the efficiency of each primer set, standard curves were constructed from serial dilutions of the purified PCR product as the template (from 2.5x10−4 to 2.5x10^-8^ ng of purified DNA).

### 2.4. Quantification of peptidase transcripts

The real-time polymerase chain reaction was carried out in a StepOne Real-Time PCR Systems (Applied Biosystems), using the Power SYBR Green Master Mix (Applied Biosystems). PCR reactions were performed in duplicate of the four biological replicates of each shrimp larval instars. The reaction mix included Power SYBR Green Master Mix at 1X, 0.5 mM of each primer, and the template equivalent to 34 ng of cDNA in a total volume of 10 μL. The reaction’s cycling profiles were as follows: 1 cycle at 95°C for 5 min and 35 cycles at 95°C for 30 s each, then the annealing temperature according to Table 1 for 30 s, and at 72°C for 30 s. For each primer set, a no template control (NTC) was included, for every case, such control showed no signal after 40 amplification cycles. The amplification data was collected using the StepOne Real-Time System software. The relative quantification of each peptidase gene expression was calculated using the 2^-ΔCT^ method using ubiquitin as internal control since ubiquitin was the most stable reference gene in the experimental conditions analyzed, as determined with the geNorm algorithm [21].

The data matrix is represented on graphic mode using a heat map where the values are expressed in different colors to represent variations of each gene expression at each one of the larval instars. The heat map was created in the online software Heatmapper [22].

### 2.5. Proteolytic activity and inhibition assays

Larvae (90 mg of each instar) were thoroughly homogenized in 330 μL of distilled water using a micro-pestle in a microtube and the cleared supernatant containing the soluble protein was obtained by centrifugation at 10,000 *g*, for 10 min at 4°C. The soluble protein concentration was quantified after Bradford [23].

The proteolytic activity near the functional pH (as reported in database BRENDA-The Comprehensive Enzyme Information System [24]) was measured in four biological replicas by continuous kinetic assays using commercially available fluorogenic substrates designed to be recognized and cleaved by specific peptidases, such substrates are short synthetic peptides attached to the fluorogenic reporter 7-aminomethyl-coumarin (AMC; Ex:360 nm, Em:440 nm) or 7-methoxycoumarin-4-acetic acid (MCA; Ex:323 nm, Em:390 nm). The assays were performed at pH 3.6, 5.0 and 6.6 in citrate-phosphate buffer and at pH 8.0 in Tris-HCl buffer in the presence of 100 mM NaCl, 1mM DTT, and 0.001% Tween; for the assays at pH 8.0, 10 mM CaCl_2_ was added, the substrate assay concentration was 2.5 μM.

Rates of hydrolysis of each substrate were recorded by measuring the increase of fluorescence in arbitrary units (relative fluorescence units, RFU) in black 96-well plates. A calibration curve was constructed by measuring the fluorescence of known concentrations of the fluorochrome and by plotting the RFU versus nanomoles of the molecule. Specific activity is expressed in nmol of AMC or MCA liberated per minute, per μg of protein. One unit of activity was defined as the release of 1 μmol of AMC or MCA per min per μg protein.

To confirm that the observed activity is specific for each enzyme, the larvae extract was pre-incubated for 30 min at room temperature with the countertype class-specific peptidase inhibitor; the aspartic peptidase inhibitor pepstatin A (1 μM final concentration), the cysteine peptidase inhibitor E-64 (30 μM final concentration) or the serine peptidase inhibitor PMSF (1 mM final concentration), and EDTA at 1 mM was used to chelate metal ions necessary for metallo-peptidase activity. The residual proteolytic activity was determined as described above, only the portion of the activity that resulted sensitive to the countertype inhibitor (the total activity minus the activity in the presence of inhibitor) was considered.

### 2.6. Statistical analysis

Data are summarized in box and whiskers plots showing the median and the min to max of four biological replicates quantified in duplicate. The statistical differences among different groups were determined by the Kruskal-Wallis test, the post hoc analysis Dunn’s Multiple Comparison Test was applied and considered as statistically different when p < 0.05, GraphPad Prism 5 (GraphPad Software, USA) was used for statistical procedures and graph plotting.

## 3. Results

### 3.1. Peptidase gene expression profile during P. vannamei larval development

**Fig 2:**
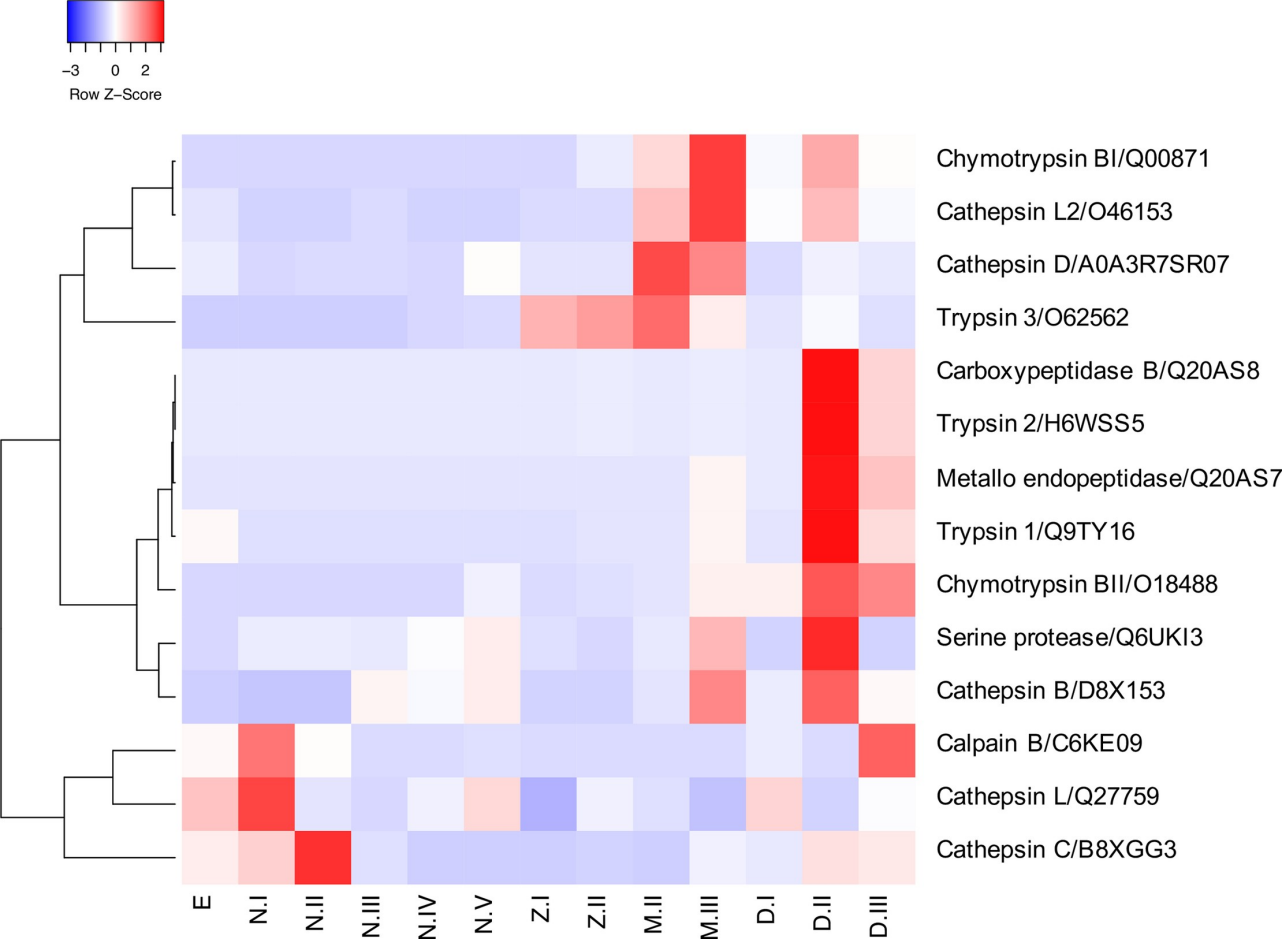
Peptidase gene expression patterns in the larval stages of *P*. *vannamei*. Heatmap colors represent relative mRNA expression as indicated in the color key. Clusters of expression patterns were calculated by hierarchical methods with Pearson's correlation similarity and average linkage distance.

Patterns of gene expression of the selected peptidases throughout the larval development of *P*. *vannamei* are readily visualized in a heat map (Fig 2). Similar expression patterns were calculated by hierarchical clustering with Pearson's correlation similarity and average linkage distance. Three main clusters are detected (upper to lower clusters): a) peptidases expressed mainly in zoeal and mysis stages, this is a diverse group comprised by four genes encoding peptidases of the serine-, cysteine- and aspartic-classes; b) peptidases highly expressed at decapodid stages, this is the larger group comprising seven genes and includes well-characterized peptidases described as food protein-digestors as trypsin 1 and 2 [25], chymotrypsin [26] and cathepsin B [27] and; c) peptidases expressed mainly at the early naupliar instars, this group comprising the cysteine peptidases Calpain B, Cathepsin L and Cathepsin C.

### 3.2. Peptidase activity profile during P. vannamei larval development

**Fig 3:**
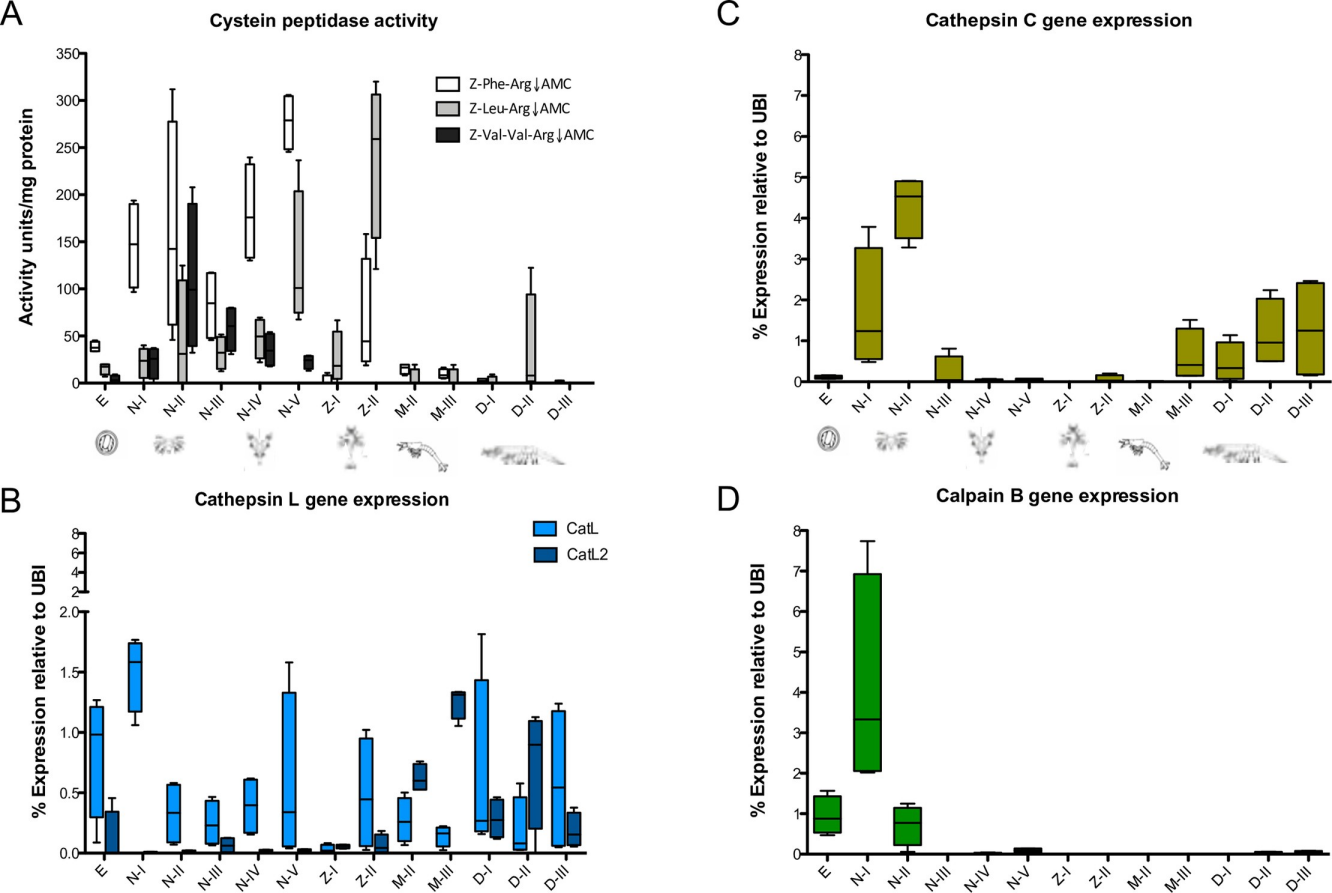
Cysteine peptidase gene expression and enzymatic activity throughout the larval development of *P*. *vannamei*. A) cysteine peptidase activity (sensitive to inhibition by E-64), B) cathepsin L and L2, C) cathepsin C, and D) calpain B gene expression.

The proteolytic activity from the four peptidase classes was assessed. To quantify the cysteine peptidase activity, three substrates were used, Z-Phe-Arg↓AMC, Z-Leu-Arg↓AMC and Z-Val-Val-Arg↓AMC (arrow indicates the site of the scissile bond); although such substrates are recognized and efficiently cleaved by crustacean cysteine peptidases [28, 29], they might be also hydrolyzed by other non-cysteine peptidases [30], therefore only the portion of the activity that resulted sensitive to the cysteine peptidase inhibitor E-64 was considered. In agreement with the cysteine peptidase gene expression observed in the heat map (Fig 2, lower cluster), peaks of activity were observed during the early larval stages (Fig 3A). Such activity can be attributed to cathepsin L (Fig 3B), cathepsin C (Fig 3C) and calpain B (Fig 3D), since those genes showed the highest expression at naupliar stages.

**Fig 4:**
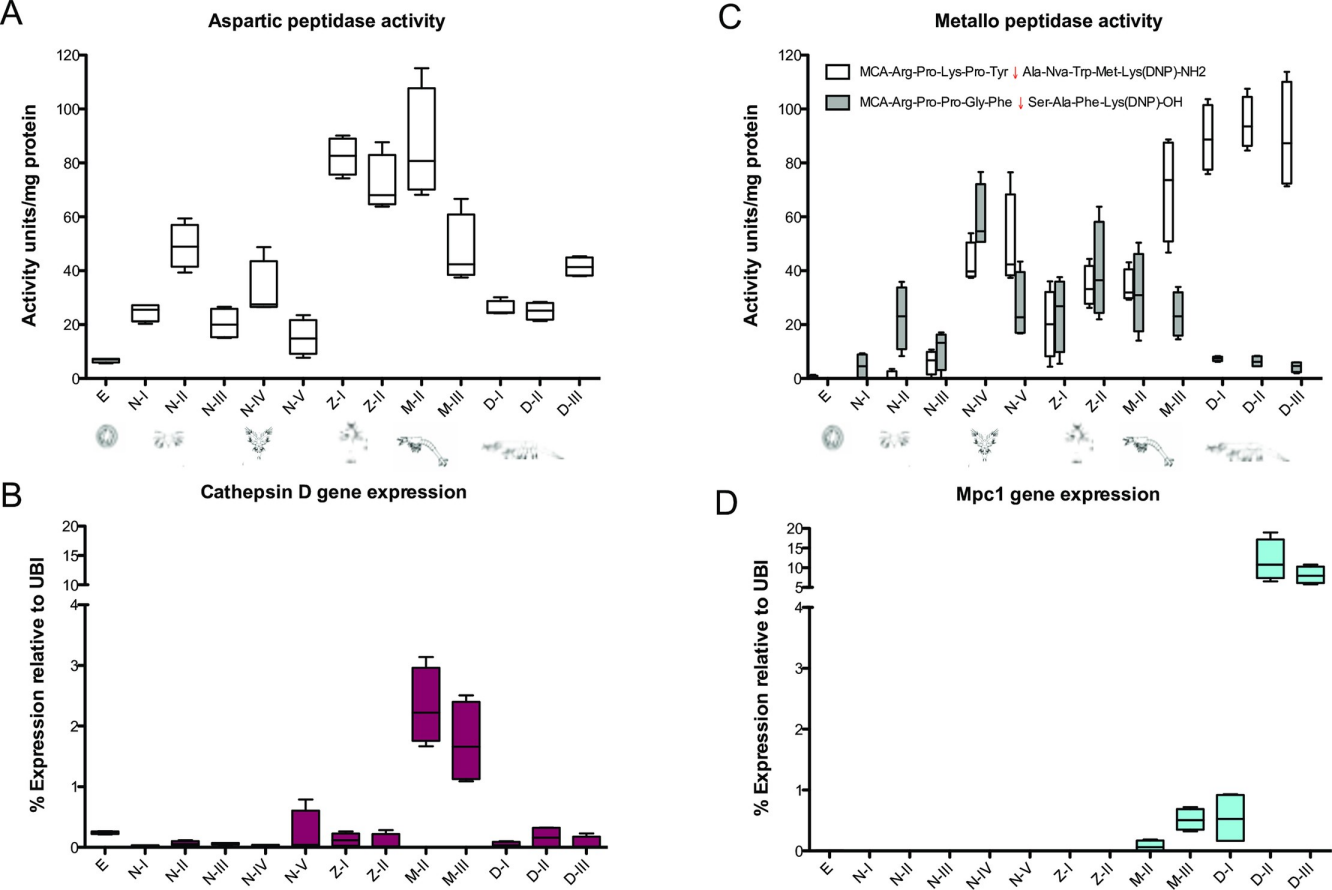
Aspartic and metallopeptidase gene expression and enzymatic activity throughout the larval development of *P*. *vannamei*. A) Aspartic peptidase activity (sensitive to inhibition by Pepstatin A), B) cathepsin D gene expression, C) Metallo peptidase activity (sensitive to inhibition by EDTA), D) Mpc1 gene expression.

Aspartic peptidase activity was assessed using the substrate MCA-Gly-Lys-Pro-Ile-Leu-Phe↓Phe-Arg-Leu-Lys(DNP)-DArg-NH2, known to be hydrolyzed by cathepsin D and cathepsin E enzymes from diverse vertebrate and invertebrate species. A generally constant amount of aspartic peptidase activity was observed during the larval development with an increase at nauplii-II substage and then at zoea and mysis stages (Fig 4A). *P*. *vannamei* expresses at least one cathepsin D transcript (GenBank: MH171099.1; UniProt: A0A3R7SR07) and our results indicate that in general, the peak of gene expression at mysis stages (Fig 4B) match with the activity found at late zoea and mysis stages. A putative second isoform is reported in GenBank (XM_027380068) which sequence is derived from a genomic assembly (BioProject: PRJNA508983), its expression was not assessed in this work, but might be responsible for the rise in activity found in naupliar instars.

A zinc metallopeptidase expressed in the digestive tissues of *P*. *vannamei* has been reported [16], this enzyme belongs to the astacin family (PFAM: PF01400) and shares 59.1% of identity with *Astacus astacus* astacin, the eponym of this peptidase family that is also a digestive enzyme [31, 32]. Astacin effectively hydrolyze peptides that are bradykinin derivates, like MCA-Arg-Pro-Pro-Gly-Phe↓Ser-Ala-Phe-Lys(DNP)-OH [33], which was used as a substrate to assess the activity of this enzyme class on developmental stages of *P*. *vannamei*. The activity against the metallo-peptidase substrate MCA-Arg-Pro-Lys-Pro-Tyr↓Ala-Nva-Trp-Met-Lys(DNP)-NH2 was also assessed (Fig 4C), and effectively hydrolyzed throughout all the larval development, especially at the early stages. An increase on substrate preference was detected in later stages where the highest values were recorded against MCA-Arg-Pro-Lys-Pro-Tyr↓Ala-Nva-Trp-Met-Lys(DNP)-NH2, the activity against this substrate coincides with the quantified metalloendopeptidase (UniProt: Q20AS7) gene expression at decapodid stages (Fig 4D), a late developmental stage with a fully mature digestive system.

**Fig 5:**
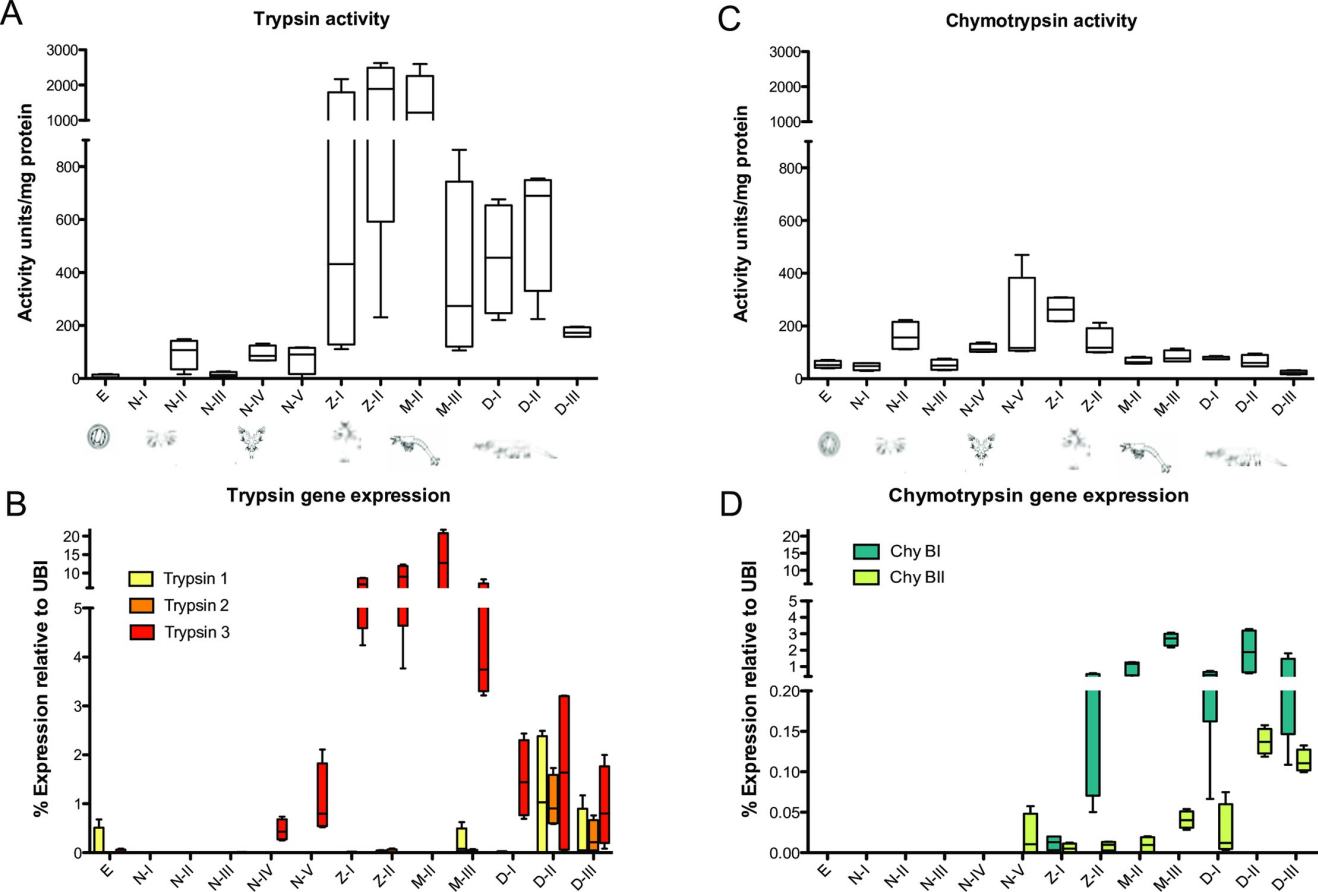
Serine peptidase gene expression and enzymatic activity throughout the larval development of *P*. *vannamei*. A) Trypsin activity (sensitive to inhibition by PMSF), B) Trypsin isoforms gene expression, C) Chymotrypsin activity (sensitive to inhibition by PMSF), and D) Chymotrypsin isoforms gene expression.

Serine peptidases are the most-studied peptidase class in *P*. *vannamei*, being trypsin and chymotrypsin the predominant peptidases from the digestive tract of penaeids [34]. Boc-Phe-Ser-Arg ↓ AMC and Suc-Ala-Ala-Pro-Phe ↓ AMC were used as substrates to quantify trypsin and chymotrypsin activity respectively (Fig 5A and 5C). Activity against trypsin substrate was by far the most abundant during the larval development with the highest values recorded during the zoea and mysis instars and a decline at decapodid instars, this activity fully matches with the expression pattern of trypsin 3 gene (UniProt: O62562) (Fig 5B). Chymotrypsin BI transcripts are abundant at mysis and decapodid instars (Fig 5D) but the activity profiles are different.

## 4. Discussion

Peptidases are known to play key functions in larval development in arthropods. Insects are particularly well-studied taxa for which the metamorphic proteolytic repertoire has been described in detail [5, 35–40]. The present study provides a comprehensive characterization of larval proteolytic profile in both transcript and activity analysis in a crustacean, the whiteleg shrimp *P*. *vannamei*.

The main protein component of yolk in *P*. *vannamei* zygotes is vitellin, a large multi-subunit lipo-glyco-caroteno-protein; its degraded products are the source of amino acids and other nutrients for development in the non-feeding larvae (see Fig 1) [41], which survival depends on the nutrient reserves of the yolk [42]. *P*. *vannamei* cysteine peptidase activity is high at the naupliar stages (Fig 3A), we presume this activity is responsible for hydrolysis of yolk proteins, as described in other arthropods [43–45]. Cathepsin L, cathepsin C and calpain are assumed to be the main enzymes responsible of the hydrolysis of vitellin given their significantly higher expression at naupliar stages (clustered in Figs 2 and 3B–3D, respectively).

Another featured function of peptidases in insect development is the transformation and removal of cuticle components during molt. Degradation of the old cuticle is accomplished by the secretion of chitinases and peptidases into the molting space [8, 46–50]. Decapod molting cycle is one of the fundamental processes for growth and development, which involves rearrangement of muscle fibers that undergo degradation of the muscular proteins actin and myosin, leading to a muscle induced atrophy, this allows limb withdrawal from narrow openings during ecdysis [51]. In *P*. *vannamei*, the formation of the exoskeleton initiates during the limb bud embryo stage [52], and is shed shortly after hatching. Several molting events accompanied by metamorphic transitions occur within a few days (11–17 days depending on the temperature), in which a new cuticle is formed and replaced cyclically at a relatively high rate to accommodate the rapidly growing larvae [42, 53, 54]. Trypsin (Fig 5A), chymotrypsin (Fig 5C), aspartic peptidase (Fig 4A) and metallo-peptitidase activity (Fig 4C) were detected in all stages of larval development, suggesting a function of these peptidases in molting-related processes such as degradation and reabsorption of proteins from the cuticle, ecdysial matter, and muscle. This has been suggested for juvenile *P*. *vannamei* in which trypsin gene expression and activity is particularly high during juvenile pre-ecdysis stages [55, 56].

A generally sustained aspartic peptidase activity during the larval development was observed in our results (Fig 4A). In insect larvae development, cathepsin D fulfills specific functions, including yolk proteins digestion [57], fat body degradation [58], and apoptotic processes linked to insect metamorphosis [5], therefore we hypothesize that *P*. *vannamei* cathepsin D is the responsible of such aspartic peptidase activity. We determined the expression pattern of a *P*. *vannamei* cathepsin D (UniProt: A0A3R7SR07), observing a generally steady expression in all stages, with a significant increase at mysis instars (Fig 4B) that clearly matches the peak of activity at late zoea and mysis stages. During *P*. *vannmaei* mysis instars, major growth and differentiation of hepatopancreatic lobes occur [59], based on our result we suggest that cathepsin D is synthesized in this tissue and could be involved in the larval morphogenesis of the midgut gland.

Metallo-peptidase activity was also assessed (Fig 4C), larval zinc metallopeptidase expression and activity had been reported mainly in the infective stages of helminths and insects [60, 61], but also as fat-body remodeling enzymes in insect larval stages [62]. The main *P*. *vannamei* metallopeptidase belongs to the astacin family and the transcript is found primarily in the digestive system [16, 63]. The expression of this enzyme was detected from late mysis stage and increasing at decapodid stages where a significant increment of activity is also observed (Fig 4D), such activity peak has been reported before [16]. *P*. *vannamei* also expresses a putative second isoform of metallo-peptidase that is deposited in the GenBank (XM_027379199), an astacin-like enzyme which sequence is derived from a genomic assembly (BioProject: PRJNA508983), its expression was not assessed in this work, the discrepancy in metallo-peptidase gene expression and activity might be an effect of the presence of an alternative isoform which might show a preference for MCA-Arg-Pro-Pro-Gly-Phe↓Ser-Ala-Phe-Lys(DNP)-OH and express steadily during the larval development.

Serine peptidase activity was detected in all larval stages. Fluctuations in trypsin and chymotrypsin peptidases expression and activity during larval stages of development is documented for *P*. *vannamei* and other crustaceans species [12, 13, 16, 64]. Trypsin (Fig 5A) and chymotrypsin activity (Fig 5C) show a significant increase at zoea stages, trypsin is by far the dominant peptidase activity in these stages and continues through the first decapodid instar. Trypsin activity profiles are consistent with the expression pattern of Trypsin 3 (UniProt: O62562) (Fig 5B), which increase in expression at zoea instars has been reported by other authors [12, 14]. Zoea is the first feeding stage during which the gut becomes complete but the rest of the digestive organs, like the cardiac and pyloric chamber and the hepatopancreas, are still undifferentiated; the zoeal gut consists of a simple tube and two pairs of caeca that fulfill digestive and absorptive functions and are the primordium to the hepatopancreas [59, 65]. During zoea, the breakdown of larval food is primarily enzymatic [42, 66], this fact explains the general increase in serine peptidase activity detected in our assays.

Juvenile and adult *P*. *vannamei* midgut gland is particularly enriched with the serine peptidases trypsin and chymotrypsin, which fulfill a digestive function breaking peptide bonds of large proteins from food into smaller peptides ready for absorption [34]. The gene expression of isoforms of trypsin and chymotrypsin was detected at decapodid stages (Fig 5B and 5C). Trypsin 1 (UniProt: Q9TY16) and 2 (UniProt: H6WSS5) are found in the digestive tract of juvenile and adult *P*. *vanamei* and reveal as 3 activity bands when analyzed by zymography [67]. Coincidentally, the gene expression of these enzymes was detected only at decapodid stages (Fig 5B), at which the digestive organs are fully operational [65]. In the heat map (Fig 2) Trypsin 1 and 2 show a similar expression pattern that clusters with hepatopancreatic enzymes described in other works by zymography (e.g. metallopeptidase and chymotrypsin BII) [16], reinforcing the functional characterization of this group as food protein digestors in decapodid and juvenile stages.

The fact that in our results peptidase gene expression and the corresponding activity profiles are not fully dependent during the biological conditions assessed (larval development) cannot be overlooked. Transcription and translation are generally regarded as independent processes due to the mechanism triggering them [68], a mismatch in enzyme gene expression and activity is common and almost considered the general rule [69], since several factors influence in the production of functional proteins. In the particular cases of peptidase gene expression during metamorphosis, this is a process highly controlled by hormonal changes in both insects and crustaceans [70, 71]. Some peptidases are classified as ecdysteroid-responsive genes, since they are known to contain ecdysone response elements in their promoters [72], and are under the control of the ecdysone cassette [73, 74]. At the protein level, molt has also been described to be controlled by peptidase inhibitors, and these molecules are considered regulators of the molting process [35].

The presence of different peptidases belonging to the same functional categories needs to be taken into account and is reported for decapods [75]. Peptidase variants or isoforms may be conducting the same physiological function at different stages, a phenomenon called isoform-switching that have has observed in crustacean larval stages [76]. The presence of class-specific peptidase activity in stages where the corresponding gene was not detected might be due to the presence of peptidase gene isoforms that are still to be annotated and evaluated.

In this study, we describe the mRNA expression and proteolytic activity patterns of several members of the cysteine- aspartic-, metallo- and serine-peptidases throughout the larval development of *P*. *vanamei*; the enzymes showed variations in expression and activity, indicating that they are controlled and play various roles in shrimp larvae development and metamorphoses like yolk degradation, cuticle transformation and degradation of unnecessary tissues. The present work contributes to a more integral perspective for understanding the developmental biology of this species, required for the technification of culture methods and proper management of natural stocks of *P*. *vannamei*. Further studies are required to describe in more detail the specific peptidase functions and interactions.

## Supporting information

S1 Table Distribution of gene expression data (mean, min and max) used to construct plots in Figs 3–5. (XLSX)

S2 Table Distribution of proteolytic activity data (mean, min and max) used to construct plots in Figs 3–5. (XLSX)
